# Air pollution and female fertility: a systematic review of literature

**DOI:** 10.1186/s12958-018-0433-z

**Published:** 2018-12-30

**Authors:** Alessandro Conforti, Marika Mascia, Giuseppina Cioffi, Cristina De Angelis, Giuseppe Coppola, Pasquale De Rosa, Rosario Pivonello, Carlo Alviggi, Giuseppe De Placido

**Affiliations:** 1 Dipartimento di Neuroscienze, Scienze Riproduttive ed Odontostomatologiche, Sezione di Ginecologia ed Ostetricia, Centro di Sterilità Università “Federico II” di Napoli, Naples, Italy; 2 I.O.S. & COLEMAN Srl, Naples, Italy; 3 Dipartimento di Medicina Clinica e Chirurgia, Sezione di Endocrinologia, Centro di Andrologia, Medicina della Riproduzione e della Sessualità Maschile e Femminile, Università “Federico II” di Napoli, Naples, Italy; 4 Centro nazionale delle ricerche, Istituto per l’Endocrinologia e l’Oncologia Sperimentale (IEOS), Naples, Italy

**Keywords:** Air pollution, IVF, Miscarriage, Live birth rate

## Abstract

Air pollution is a cause of concern for human health. For instance, it is associated with an increased risk for cancer, cardiovascular and respiratory disorders. In vitro and in vivo studies suggested that air pollutants could act as endocrine disruptors, promote oxidative stress and exert genotoxic effect. Whether air pollution affects female infertility is under debate. The aim of the present study was to conduct a systematic review of studies that evaluated the impact of air pollution on female infertility. We systematically searched the MEDLINE (PubMed) and SCOPUS databases to identify all relevant studies published before October 2017. No time or language restrictions were adopted, and queries were limited to human studies. We also hand-searched the reference lists of relevant studies to ensure we did not miss pertinent studies. The risk of bias and quality assessment of the studies identified were performed using the Newcastle-Ottawa Scale. Primary outcomes were conception rate after spontaneous intercourse and live birth rate after in vitro fertilization (IVF) procedures. Secondary outcomes were first trimester miscarriage, stillbirths, infertility, number of oocytes and embryo retrieved. Eleven articles were included in the analysis. We found that in the IVF population, nitrogen dioxide and ozone were associated with a reduced live birth rate while particulate matter of 10 mm was associated with increased miscarriage. Furthermore, in the general population, particulate matter of 2.5 mm and between 2.5 and 10 mm were associated with reduced fecundability, whereas sulfur dioxide_,_ carbon monoxide and nitrogen dioxide might promote miscarriage and stillbirths. The main limitation of our findigns resides in the fact that the desegn of studies included are observational and retrospective. Furthermore, there was a wide heterogenity among studies. Although larger trials are required before drawing definitive conclusions, it seems that air pollution could represent a matter of concern for female infertility.

**Electronic supplementary material:**

The online version of this article (10.1186/s12958-018-0433-z) contains supplementary material, which is available to authorized users.

## Introduction

Female infertility has increased in recent years [1]. It was estimated that this condition affects 1 in seven couples in developed countries [2]. Most cases of female infertility are related to specific disorders, namely, ovulatory disorders, endometriosis, chromosomal abnormalities and male factors [3–7]. There is also evidence that air pollutant could play a role in the pathogenesis of female infertility [8–10]. Air pollution appears to be a cause of concern for human health. For instance, it has been associated with an increased risk of cancer [11], and cardiovascular [12] and respiratory disorders in adults and children [13, 14]. In addition, air pollutants have been associated with adverse perinatal outcomes [15, 16].

Anthropogenic activities, namely traffic, industrial facilities and combustion of fossil fuels, which are particularly intense in large cities and in proximity of farms, are the main sources of health-related air pollutants. Air pollutants are in four main categories: gaseous pollutants (sulfur dioxide [SO_2_], nitrate oxide [NO_2_] and carbon monoxide [CO]), organic compounds (organic solvents or dioxins), heavy metals (lead and copper) and particulate matter (PM_10_ PM_2.5-10_and PM_2.5_) [17]. Ingestion and inhalation are the most common routes of exposure [17]. Ingestion is also facilitated by the fact that air pollution contributes to the contamination of food and water [18]. Some air pollutants, namely Cu, Pb and diesel exhaust seem to exert endocrine activity [19] that could affect female reproduction. Moreover, these endocrine “disruptors” exert estrogenic, antiestrogenic and antiandrogenic activity and some could interfere with the thyroid axis and influence metabolic disorders, such as insulin resistance and obesity, which are strictly related to infertility [20–22]. The increase in female infertility seems to parallel the increase in toxic emissions, which suggests that the impact of air pollution on human health could increase in the next years [23, 24]. In an attempt to summarize current evidence, we carried out a systematic review of studies devoted to the impact of air pollutions on female infertility.

## Material and methods

### Protocol and eligibility criteria

The present study was exempt from institutional and ethics board approval because it did not involve human intervention. We adhered to the Preferred Reporting Items for Systematic Reviews and Meta-Analyses (PRISMA) guidelines [25]. The selection criteria are described according to PICO (Patients, Intervention, Comparison, and Outcomes). In detail, we evaluated fertility outcomes in women on reproductive age (in the general and IVF populations) in relation to exposure to air pollutants (Additional file 1: Table S1).

### Search strategy

We conducted a systematic search using MEDLINE (PubMed) and SCOPUS databases to identify all relevant studies published before October 2017. Combinations of the following keywords and MESH search terms were used: “air pollutants” AND (“miscarriage” OR “embryo” OR “pregnancy” OR “IVF OR “fecundability” OR “infertility” OR “menstrual disorders”). No time or language restrictions were adopted, and queries were limited to human studies. We also hand-searched reference lists of relevant studies to ensure we did not miss pertinent studies.

### Selection of studies

Four reviewers (G.C., M.M., G.CO and P.D.) independently evaluated titles and abstracts. Duplications were removed using Endnote online software and manually. Disagreements were resolved by discussion with a third authors (A.C. and C.D.), and if required, with the involvement of the most experienced authors (R.P.,C.A., G.D.). Articles were included only if they appeared in peer-reviewed journals. Case series, case reports, book chapters, congress abstracts and grey literature [26], which includes a range of documents not controlled by commercial publishing organization, were not included.

### Data extraction

Data were extracted independently by four reviewers (G.C., M.M., G.CO and P.D.) using predefined data fields, including study quality indicators. Discrepancies were resolved by discussion with the senior authors (R.P., C.A. and G.D.).

### Risk of bias, summary measures and synthesis of the results

The risk of bias and quality assessment of the included studies were performed adopting the Newcastle-Ottawa Scale (NOS) [27]. Four authors (A.C, C.D., G.C. and P.D.) independently assessed the risk bias for each study. The senior authors (R.P., C.A. and G.D.) resolved conflicts. The NOS score was used to evaluate the studies included, and judgment on each one was passed according to three issues: selection of the study group, comparability between groups, and ascertainment of exposed/not exposed cohorts. Primary outcomes were conception rate after spontaneous intercourse and live birth rate after IVF procedures. Secondary outcomes were first trimester miscarriage, stillbirths, infertility, number of oocytes retrieved and embryos transferred.

## Results

### Study selection and characteristics

**Fig. 1:**
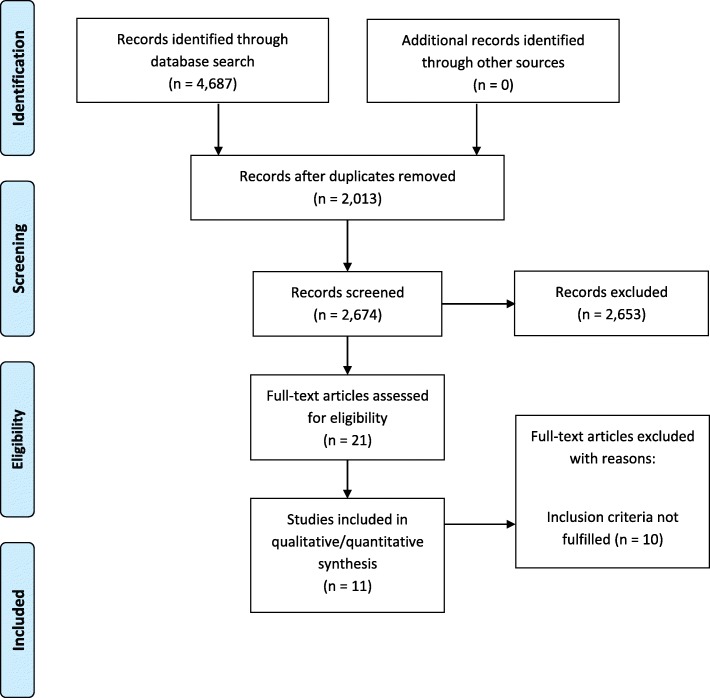
Flow chart

A total of 4687 items were identified (Pubmed 2834 and Scopus 1853). A total of 2013 duplicates were removed manually and using the EndNote online library. The titles and abstracts of 2674 papers were scrutinized and 21 full papers were assessed for eligibility. Ten papers were excluded because they did not fulfill the inclusion criteria. Eleven articles were included in the analysis (Fig. 1). The characteristics of the studies included in the present study are reported in Table 1.

**Table 1:** Characteristics, findings and risk of bias of included studies

Author, Year, (ref)	Study design	Population Country Individuals		Pollutants	Exposure	Confounders adjusted for	Significant effect size:	Effect size	95% CI	NOS
Dejmek et al. (2000) [33]	Retrospective cohort	Czech Republic	2585 (General population)	SO2	Monitoring station obtained from by US EPA (Air Quality System)	Maternal age; parity; conception; seasonality; currently married; temperature average; temperature maxima; signal; year; season; epidemiological situation	Conception in the first unprotected menstrual cycle	OR = 0.57 SO2 levels (40-80 μg/m^3^)	0.37–0.88	7
								OR = 0.49 SO2 levels (≥ 80 μg/m^3^)	0.29–0.81	
Sallmen et al. (2008) [34]	Retrospective cohort	Portugal	406 (General population)	Solvents used in shoe manufacturing (N-hexane and hexane isomers; Toluene; Methyl ethyl ketone; Acetone; Ethyl acetate; dichloromethane)	Air sampling was performed in the personal breathing zones of the exposed women, spanning roughly an 8-h work shift.	Female age; Last method of contraception; Age at menarch; Regularity of menstrual cycle; Male smoking; Female and male use of alcohol; Male exposure to metal dusts or fumes; Male exposure to engine exhausts.	Fecundability density ratio (low exposure to solvents)	FDR = 0.55	0.40–0.74	7
							Fecundability density ratio (high exposure to solvents)	FDR = 0.70	0.52–0.94	
Green et al. (2009) [35]	Prospective cohort	USA	4979 (General population)	Traffic pollutants: NO2; O3; PM 2.5; PM 10, CO2 CH4, CO, H2S, NMHC NMOC; SO2; sulphur; THC	Traffic exposure were constructed using annual average daily traffic (AADT) counts near each residence and distance from residence to major roads	Maternal age, race, employment status, stressful life events and maternal smoking	Spontaneous abortion Maximum daily traffic within 50 m^2^			7
							> 90 centile	OR = 1.18	0.87–1.60	
							> 90 centile (African American)	OR = 3.11	1.26–7.66	
							> 90 centile (non smokers)	OR = 1.47	1.07–2.04	
Mohorovic et al. (2010) [36]	Prospective cohort	Croatia	260 (General population)	Coal combustion (NO2; CO2; CO; other products)	Monitoring station (Labin meteorological station)	Crude data	Spontaneous abortion	OR = 2.99	0.91–9.80	5
Perin et al. (2010) [31]	Retrospective cohort	Brazil	348 (IVF women)	PM10	PM10 concentrations taken from 14 monitoring stations categorized into quartiles (Q1-Q4).	Ovarian response patterns to gonadotrophins, exposure, patient’s age, and the year of IVF treatment	Miscarriage in IVF women (> 56.72 µg/m3)	OR = 5.05	1.04–25.51	8
							Live birth rates (> 56.72 µg/m3)	OR = 1.71	0.72–4.09	
Perin et al. (2010) [32]	Retrospective cohort	Brazil	177 (IVF women) 354 (General population)	PM10	PM10 concentrations taken from 14 monitoring stations categorized into quartiles (Q1-Q4).	Ovarian response patterns to gonadotrophins, exposure, patient’s age, and the year of IVF treatment	Miscarriage in general population (> 56.72 µg/m3)	OR = 2.72	1.51–4.89	7
							Miscarriage in IVF women (> 56.72 µg/m3)	OR = 2.32	1.00–5.43	
Legro et al. (2010) [28]	Retrospective cohort	USA	7403 (IVF women)	PM2.5 PM10 SO2 NO2 O3	Monitoring station obtained from by US EPA (Air Quality System)	Age, IVF center and the year and season of oocyte retrieval	Live Birth Rate NO2 (after embryo transfer) O3 (after embryo transfer**)**:	OR = 0.76 OR = 0.62	0.66–0.86 0.48–0.81	9
							Pregnancy rate PM2.5 (during embryo culture)	OR = 0.94	0.82–0.99	
Faiz e al. (2012) [30]	Retrospective cohort	USA	343,077 (General population)	PM 2.5 SO2 NO2 CO	Central monitoring station monitored by Agency Air Quality System	Maternal age; Race/Ethnicity; Educational level; Prenatal care; Smoking; Neighborhood socioeconomic status; Calendar year; month of conception and; mean temperature	Stillbirths NO2 (first trimester) SO2 (first trimester) CO (second trimester)	OR = 1.16 OR = 1.13 OR = 1.14	1.03–1.31 1.01–1.28 1.06–1.24	8
Slama et al. [29]	Retrospective cohort	Czech Republic	1916 (General population)	SO2, PM2.5, NO2, O3, carcinogenic PAHs	Central monitoring station	Maternal age, smoke habits and alcohol consumption before pregnancy,maternal education, marital status, BMI	Fertility rate PM2.5 NO2	FR = 0.78 FR = 0.72	0.65–0.94 0.53--0.97	9
Nieuwenhuijsen et al. (2014) [9]	Cross-sectional	Spain	not available (General population)	PM10 PM2.5 PMcoarse fraction NO2 NOx O3 PM2.5 adsorbance	Land use regression developed in the European Study of Cohorts for Air Pollution Effects	Socioeconomic status, ethnicity, age, educational level	Fertility rate PM coarse fraction	FR = 0.88	0.83–0.94	7
Mahalingaiah et al. (2016) [8]	Prospective cohort	USA	36,294 (General population)	PM 10, PM 2.5, PM 2.5–10	USEPA Air Quality System	Age, smoking status, Race, BMI, parity, rotation shift work, oral contraception use, diet, Census tract level median income and median home value	Hazard ratio of primary and secondary infertility Living closer a major roads	HR = 1.11	1.02–1.20	9

### Risk of bias

The risk of bias was evaluated with the NOS score and is reported in Table 1.

### Summary of results

We summarized our findings considering per each pollutant both IVF women and reproductive age women in general population (Table 2).

**Table 2:** Synthesis of results

Type of Pollutant	Population	Effect
NO_2_	IVF	Lower live birth rates
	General population	Higher miscarriage rate
CO	General population	Higher stillbirth in second and third trimester
O_3_	IVF	Lower live birth rates
PM_2.5_	IVF	Lower pregnancy rates
	General population	Reduced fecundability ratio
PM_10_	IVF	Higher miscarriage rate
	General population	Higher miscarriage rate
PM_2.5–10_	General population	Reduced fertility rate
SO_2_	IVF	No effect
	General population	Higher early miscarriage and third trimester still births. Reduced conception rate
Traffic pollutants	General population	Higher miscarriage rate; Higher infertility rates.
Coal combustion products	General population	Higher trend of miscarriage

### NO_2_

#### IVF cycles

Increases in NO_2_ concentrations were significantly associated with a lower live birth rate especially from embryo transfer to pregnancy test (OR 0.76, 95% CI 0.66–0.86, per 0.01 ppm increase) [28]. No effect on the number of oocytes retrieved or embryo transferred was observed [28].

#### General population

In a cross-sectional study involving women of reproductive age between 15 and 40 years, the fertility rate was not significantly associated with NO_2_ exposure (OR 0.97, 95% CI 0.94–1.003) [9]. In contrast, another retrospective cohort study, showed that there was a significant decreased fecundability ratio per each increase of 10 μg/m^3^ NO_2_ exposure (OR 0.72, 95% CI 0.53–0.97) [29]. Miscarriage rate was significantly increased in women exposed to NO_2_ compared to not exposed group (OR 1.16, 95% CI 1.01–1.28, per each 10-ppb increase in NO_2_ concentration) [30].

### CO

#### General population

Exposure to CO was significantly associated with stillbirth in the second (OR = 1.14, 95% CI: 1.01, 1.28) and third trimester (OR = 1.14, 95% CI: 1.06, 1.24) [30]. No significant association with first trimester miscarriage was reported (OR = 1.14, 95% CI 0.98, 1.32) [30].

### O_3_

#### IVF-cycles

A detrimental effect was observed in terms of live birth rate in women exposed to O_3_ from embryo transfer to date of live birth (OR 0.62, 95% CI 0.48–0.81, per 0.02 ppm increase) [28]. No effect on the number of oocytes retrieved or embryo transferred was observed [28].

#### General population

Only one study assessed the fecundability rate in the general population but no difference was reported between exposed and unexposed group [29].

### PM_2.5_

#### IVF cycles

Exposure to PM_2.5_ during embryo culture was associated with a decreased conception rate (OR 0.90, 95% CI 0.82–0.99, per 8 μg/m^3^ increase) but not with live birth rates [28]. No effect on the number of oocytes retrieved or embryo transferred was observed [28].

#### General population

Multivariate hazard ratio (HR) analysis did not reveal any association with infertility considering 2 years average exposure (HR 1.09, 95% CI 0.77–1.55), 4 years average exposure (HR 0.91, 95% CI 0.78–1.05) and cumulative average exposure (HR 1.05, 95% CI 0.93–1.20) [8]. Consistently, in another trial multivariate analysis did not reveal any association with fertility rate [9]. On the other hand, The adjusted fecundability ratio was significantly decreased with each increase of 10 units (0.78, 95% CI 0.65–0.94) [29]. No statistically significant difference was observed in terms of late (second and third trimester) or early miscarriage (first trimester) [30].

### PM_2.5–10_

#### General population

Multivariate HR analysis did not reveal any association between infertility and PM_2.5–10_ considering 2-year average exposure (HR 1.10, 95% CI 0.98–1.23), 4 year average exposure (HR 1.05, 95% CI 0.93–1.19) and cumulative exposure (HR 1.10, 95% CI 0.99–1.22) [8]. Conversely, another study reported a significant reduction of spontaneous fertility rate in women exposed to PM_2.5–10_ (incidence risk ratio: 0.88, 95% CI 0.84, 0.94) [9].

### PM_10_

#### IVF cycles

No significant effect was observed in terms of live birth rate, number of oocytes retrieved or embryos transferred in exposed women undergoing their first IVF cycle [28]. Furthermore, no significant effect was observed in the amount of gonadotropin used, number of oocytes retrieved, number of MII oocytes, embryo quality, clinical and live birth rate [26, 31]. A higher risk of miscarriage was observed in women with a higher exposure to PM_10_ (> 56.72 μg/m^3^) comparing with those exposed to lower amount of PM_10_ (≤ 56.72 μg/m^3^) (OR 5.05 95% CI 1.04–25-51) [31].

#### General population

Multivariate adjusted HR analysis per year did not reveal any association with infertility considering 2 years average exposure (HR 1.04, 95% CI 0.96–1.11), 4 years average exposure (HR 0.99, 95% CI 0.91–1.08) and cumulative average exposure (HR 1.06, 95% CI 0.99–1.13) infertility [8]. Multivariate incidence risk (IRR) ratio adjusted did not reveal any association between PM_10_ exposure and fertility rate (IRR 0.99, 95% CI 0.96–1.02) [9]. A significant association with early miscarriage was observed in women exposed to over 56.72 µg/m3. [32].

### SO_2_

#### IVF cycles

Exposure to SO_2_ did not significantly affect birth rate, number of oocytes retrieved or embryos transferred in women undergoing their first IVF cycle [28].

#### General population

No differences in terms of adjusted fecundability rate was observed per an increase of 10 units in the SO_2_ pollutant levels [29]. Conversely, in another study, the fecundability in the first unprotected menstrual cycle was significantly reduced only in couples exposed in the second month before conception to the following SO2 levels: 40–80 μg/m^3^ (OR 0.57, 95% CI 0.37–0.88); ≥ 80 μg/m^3^ (OR 0.49, 95% CI 0.29–0.81) [33]. The adjusted odds of miscarriage were significantly associated to SO_2_ exposure (OR 1.13, 95% CI 1.01–1.28 per each 3 ppb increase in concentration) [30].

### Organic solvents

#### General population

Female exposure to air contaminated with organic solvents (hexane and hexane isomers, toluene, methyl ethyl ketone, acetone, ethyl acetate, isopropyl alcohol and dichloromethane, n-hexane, hexane isomers and toluene) was associated with reduced fecundability density ratio (FDR = 0.55, 95% CI 0.40–0.74) for low exposure (exposure index 0.01–0.14), and for high exposure (exposure index > 0.14), (FDR = 0.70, 95% CI 0.52.0.94). Moreover, exposure for less than 6 years was more strongly associated with reduced FDR in both low (FDR = 0.50, 95% Cl 0.30 to 0.83) and high exposure groups (FDR = 0.50, 95% CI 0.28–0.90) [34].

### Traffic pollutants

#### General population

In a large cohort study involving 4979 women, traffic pollutants were associated with an increased but not with significant risk of miscarriage rate among women exposed to a maximum annual average of traffic pollutants within 50 m (AOR 1.18 95%, CI 0.87–1.60). A significant association was observed in a subgroup analysis involving African Americans (AOR = 3.11; 95% CI, 1.26–7.66) and nonsmokers (AOR = 1.47; 95% CI, 1.07–2.04) [35]. In another large cohort study, women living closer to a major road had a higher risk of infertility than did women living far from a major road (HR, 1.11 95% CI: 1.02–1.20) [8].

### Coal combustion pollutants

#### General population

In a small prospective study of 260 women, the miscarriage rate was higher, albeit not significantly, in women exposed to coal combustion pollutants than in non-exposed women (OR 2.99, 95% CI 0.91–9.80) [36] .

## Discussion

Only 11 studies have evaluated the potential effect of air pollutants on female reproduction.. In the IVF context, NO_2_ and O_3_ were associated with impaired live-birth rates. In addition, exposure to high levels of PM_10_ (> 56.72 μg/m^3^) resulted in an increased miscarriage rate after IVF procedures. Consistently, no study reported a significant effect on other quantitative (i.e. number of oocytes retrieved, number of embryos transferred, and consumption of gonadotropin) and qualitative (embryo quality, and number of MII oocytes) IVF outcomes [28, 31, 32]. In natural conception, reduced fecundability was associated with solvents and SO_2_ [33, 34]. Notably while abortion rate was associated with traffic pollutants [8, 35], and in particular SO_2_ and NO_2_ [30], no clear relation to coal combustion pollutants emerged [36]. Contrasting findings between infertility and PM_2.5–10_ were reported [8, 9].

Only three retrospective studies evaluated the effects of air pollution on IVF [28, 31, 32]. Although Legro and colleagues studied a large IVF population, the heterogeneity of IVF protocols and the lack of information about male partners represent two important limitation factors [28]. Moreover, the two studies conducted by Perin et al., are limited by the fact that only one pollutant was investigated and by the low number of cases enrolled [31, 32].

Eight studies have been conducted on the general population. Of the three prospective studies, the one by Mahalingaiah et al. is the largest (more than 36,000 patients) and has the highest qualitative NOS score [8]. The quality of evidence was lowest in the study by Mohorovic et al. as was the number of observations, and the authors did not report effect size for each air pollutant separately [36]. The same weakness emerges in the Green et al. paper, which however analyzed such important factors as work exposure, residential history and employment status of the population studied [35]. Of the five retrospective studies conducted to-date, the quality of evidence is highest in two large studies conducted by Faiz and colleagues [30] and by Slama and colleagues [29] demonstrating that air pollutants significantly affect fertility and stillbirths rates. The remaining three retrospective studies have several limitations, namely a paucity of data regarding the population studied [9], a low number of pollutant analyzed [33] and the methods adopted to assess exposure [34].

The relationship between air pollutants and spontaneous fertility was first observed in an animal model [37]. In detail, Mohallem et al. observed an increased implantation failure rate and a significant reduction of births in mice exposed to polluted city air compared to non-exposed mice [38]. Similarly, Veras et al. found significantly fewer antral follicles and a lower fertility index in mice exposed to traffic pollutants versus non-exposed mice [39].

The effect of air pollutants on human spermatogenesis has also been investigated [40–43]. The largest study, conducted by Hammoud et al., reported that PM_2.5_ exposure negatively correlated with sperm morphology and motility [40]. The negative effect of particulate matter was confirmed in a recent prospective cohort study that identified a significant association between PM_10_ and PM_2.5_ and sperm chromosomal abnormalities (i.e. disomy Y and disomy chromosome 21) [44].

The mechanism underlying the effect of air pollutants on female fertility is still a matter of debate. Several pathogenetic mechanisms have been proposed. Firstly, it was hypothesized that air pollutants could mimic the effect of androgens and estrogens in humans [45]. These endocrine-disrupting properties could exert their effect by interacting with nuclear receptor, the estrogen or androgen repertory or by interacting with specific targets in cytosol thus resulting in activation of the /Ras/Erk pathway [46]. Others have suggested that air pollutants could promote oxidative stress and inflammatory processes [17]. In this sense, we recently demonstrated that the addition of anti-oxidant factors to ovarian stimulation could improve reproductive outcome in women with polycystic ovarian syndrome [47]. However, whether antioxidant products could mitigate the effect of air pollutants on IVF outcomes remains to be determined. Finally, it has been suggested that air pollutants could exert a genotoxic effect. For instance, increased sperm DNA fragmentation was associated with exposure to elevated air pollution levels (at or above the upper limit of US air quality standards) [48]. Furthermore, DNA methylation seems to be significantly influenced by air pollutants [49]. Indeed, in a recent study of 777 men, an increase in air pollutant concentrations was significantly associated with F3, ICAM-1, and TLR-2 hypomethylation, and IFN-γ and IL-6 hypermethylation [50].

Our review has several limitations. First, most of the studies included in our analysis are observational and retrospective, and hence more prone to bias. Second, exposure ascertainment was heterogeneous among studies. Most of the trials assessed air quality using a specific air monitoring station, others estimated exposure according to proximity to the potential source [8, 35, 36]. In addition, the reference levels of each pollutant varied significantly among studies. Lastly, the populations investigated as well as the definitions used to assess infertility and miscarriage were also heterogeneous. These factors render a meta-analytic and quantitative approach to this issue challenging.

In conclusion, our meta-analysis suggests there is a close association between female infertility and air pollution. However, a more robust meta-analytic approach is required before any definitive conclusion can be reached.

## Additional file

Additional file 1: **Table S1.** Selection criteria according to PICO questions. (DOCX 14 kb)

## Abbreviations

AOR: Adjusted Odds ratio
BMI: Body mass index
CO: Carbon monoxide
Cu: Copper
ERK: Extracellular Signal-regulated Kinase
F3: Tissue factor
FDR: Fecundability density ratio
HR: Hazard ratio
ICAM-1: Intercellular adhesion molecule 1
IL-6: Interleukin-6
INF-γ: Interferon gamma
IRR: incidence risk ratio
NO_2_: Nitrogen dioxide
NMHC: Non-Methane hydrocarbons
NMOC: Non-Methane organic compounds
NOS: Newcastle-Ottawa Scale
O_3_: Ozone
OR: Odds ratio
Pb: Lead
PM_10_: Particulate matter of 10 μm
PM_2.5_: Particulate matter of 2.5 μm
PM_2.5–10_: Particulate matter of 2.5–10 μm (coarse fraction).
RAS: Signal transducing protein
SO_2_: Sulphur dioxide
SRC: Signal transducing protein tyrosine kinase
THC: Total hydrocarbons
TLR-2: Toll-like receptor 2

## References

[1] Talmor A, Dunphy B (2015). Female obesity and infertility. Best Pract Res Clin Obstet Gynaecol.

[2] Healy DL, Trounson AO, Andersen AN (1994). Female infertility: causes and treatment. Lancet (London).

[3] ASRM (2006). Effectiveness and treatment for unexplained infertility. Fertil Steril.

[4] National Collaborating Centre for Ws, Children's H. National Institute for Health and Clinical Excellence: Guidance (2013). Fertility: Assessment and Treatment for People with Fertility Problems.

[5] Rocca ML, Venturella R, Mocciaro R, Di Cello A, Sacchinelli A, Russo V (2015). Polycystic ovary syndrome: chemical pharmacotherapy. Expert Opin Pharmacother.

[6] Alviggi C, Carrieri PB, Pivonello R, Scarano V, Pezzella M, De Placido G (2006). Association of pelvic endometriosis with alopecia universalis, autoimmune thyroiditis and multiple sclerosis. J Endocrinol Investig.

[7] D'Argenio V, Nunziato M, D'Uonno N, Borrillo F, Vallone R, Conforti A (2017). Indications and limitations for preimplantation genetic diagnosis. Biochimica Clinica.

[8] Mahalingaiah S, Hart JE, Laden F, Farland LV, Hewlett MM, Chavarro J (2016). Adult air pollution exposure and risk of infertility in the Nurses’ Health Study II. Human Reprod.

[9] Nieuwenhuijsen MJ, Basagana X, Dadvand P, Martinez D, Cirach M, Beelen R (2014). Air pollution and human fertility rates. Environ Int.

[10] Alviggi C, Guadagni R, Conforti A, Coppola G, Picarelli S, De Rosa P (2014). Association between intrafollicular concentration of benzene and outcome of controlled ovarian stimulation in IVF/ICSI cycles: a pilot study. J Ovarian Res.

[11] Loomis D, Grosse Y, Lauby-Secretan B, El Ghissassi F, Bouvard V, Benbrahim-Tallaa L (2013). The carcinogenicity of outdoor air pollution. Lancet Oncol.

[12] Shah AS, Langrish JP, Nair H, McAllister DA, Hunter AL, Donaldson K (2013). Global association of air pollution and heart failure: a systematic review and meta-analysis. Lancet.

[13] Tomaskova H, Tomasek I, Slachtova H, Polaufova P, Splichalova A, Michalik J (2016). PM10 air pollution and acute hospital admissions for cardiovascular and respiratory causes in Ostrava. Cent Eur J Public Health.

[14] Li S, Williams G, Jalaludin B, Baker P (2012). Panel studies of air pollution on children's lung function and respiratory symptoms: a literature review. J Asthma.

[15] Dadvand P, Parker J, Bell ML, Bonzini M, Brauer M, Darrow LA (2013). Maternal exposure to particulate air pollution and term birth weight: a multi-country evaluation of effect and heterogeneity. Environ Health Perspect.

[16] Brauer M, Lencar C, Tamburic L, Koehoorn M, Demers P, Karr C (2008). A cohort study of traffic-related air pollution impacts on birth outcomes. Environ Health Perspect.

[17] Kampa M, Castanas E (2008). Human health effects of air pollution. Environ Pollut.

[18] Thron RW (1996). Direct and indirect exposure to air pollution. Otolaryngol Head Neck Surg.

[19] Ho SM, Johnson A, Tarapore P, Janakiram V, Zhang X, Leung YK (2012). Environmental epigenetics and its implication on disease risk and health outcomes. ILAR J.

[20] Santos-Silva Ana Paula, Andrade Marcelle Novaes, Pereira-Rodrigues Paula, Paiva-Melo Francisca Diana, Soares Paula, Graceli Jones Bernardes, Dias Glaecir Roseni Mundstock, Ferreira Andrea Claudia Freitas, de Carvalho Denise Pires, Miranda-Alves Leandro (2018). Frontiers in endocrine disruption: Impacts of organotin on the hypothalamus-pituitary-thyroid axis. Molecular and Cellular Endocrinology.

[21] Alviggi C, Conforti A, Rosa PD, Strina I, Palomba S, Vallone R (2017). The distribution of stroma and antral follicles differs between insulin-resistance and hyperandrogenism related polycystic ovarian syndrome. Front Endocrinol.

[22] Soave I, Caserta D, Wenger JM, Dessole S, Perino A, Marci R (2015). Environment and endometriosis: a toxic relationship. Eur Rev Med Pharmacol Sci.

[23] Lelieveld J, Evans JS, Fnais M, Giannadaki D, Pozzer A (2015). The contribution of outdoor air pollution sources to premature mortality on a global scale. Nature.

[24] Giannadaki D, Lelieveld J, Pozzer A (2016). Implementing the US air quality standard for PM2.5 worldwide can prevent millions of premature deaths per year. Environ Health.

[25] Moher D, Altman DG, Liberati A, Tetzlaff J. PRISMA statement. Epidemiology (Cambridge, Mass). 2011;22:128; author reply. 10.1097/EDE.0b013e3181fe7825 21150360

[26] Adams J, Hillier-Brown FC, Moore HJ, Lake AA, Araujo-Soares V, White M (2016). Searching and synthesising ‘grey literature’ and ‘grey information’ in public health: critical reflections on three case studies. Syst Rev.

[27] Wells G, Shea B, O’connell D, Peterson J, Welch V, Losos M, et al. Quality Assessment Scales for Observational Studies. Wiley: Ottawa Health Research Institute; 2004.

[28] Legro RS, Sauer MV, Mottla GL, Richter KS, Li X, Dodson WC (2010). Effect of air quality on assisted human reproduction. Hum Reprod.

[29] Slama R, Bottagisi S, Solansky I, Lepeule J, Giorgis-Allemand L, Sram R (2013). Short-term impact of atmospheric pollution on fecundability. Epidemiology.

[30] Faiz AS, Rhoads GG, Demissie K, Kruse L, Lin Y, Rich DQ (2012). Ambient air pollution and the risk of stillbirth. Am J Epidemiol.

[31] Perin PM, Maluf M, Czeresnia CE, Januario DA, Saldiva PH (2010). Impact of short-term preconceptional exposure to particulate air pollution on treatment outcome in couples undergoing in vitro fertilization and embryo transfer (IVF/ET). J Assist Reprod Genet.

[32] Perin PM, Maluf M, Czeresnia CE, Nicolosi Foltran Januario DA, Nascimento Saldiva PH (2010). Effects of exposure to high levels of particulate air pollution during the follicular phase of the conception cycle on pregnancy outcome in couples undergoing in vitro fertilization and embryo transfer. Fertil Steril.

[33] Dejmek J, Jelinek R, Solansky I, Benes I, Sram RJ (2000). Fecundability and parental exposure to ambient sulfur dioxide. Environ Health Perspect.

[34] Sallmen M, Neto M, Mayan ON (2008). Reduced fertility among shoe manufacturing workers. Occup Environ Med.

[35] Green RS, Malig B, Windham GC, Fenster L, Ostro B, Swan S (2009). Residential exposure to traffic and spontaneous abortion. Environ Health Perspect.

[36] Mohorovic L, Materljan E, Brumini G (2010). Consequences of methemoglobinemia in pregnancy in newborns, children, and adults: issues raised by new findings on methemoglobin catabolism. J Matern Neonatal Med.

[37] Carre J, Gatimel N, Moreau J, Parinaud J (2017). Does air pollution play a role in infertility?: a systematic review. Environ Health.

[38] Mohallem SV, de Araujo Lobo DJ, Pesquero CR, Assuncao JV, de Andre PA, Saldiva PH (2005). Decreased fertility in mice exposed to environmental air pollution in the city of Sao Paulo. Environ Res.

[39] Veras MM, Damaceno-Rodrigues NR, Guimaraes Silva RM, Scoriza JN, Saldiva PH, Caldini EG (2009). Chronic exposure to fine particulate matter emitted by traffic affects reproductive and fetal outcomes in mice. Environ Res.

[40] Hammoud A, Carrell DT, Gibson M, Sanderson M, Parker-Jones K, Peterson CM (2010). Decreased sperm motility is associated with air pollution in Salt Lake City. Fertil Steril.

[41] Guven A, Kayikci A, Cam K, Arbak P, Balbay O, Cam M (2008). Alterations in semen parameters of toll collectors working at motorways: does diesel exposure induce detrimental effects on semen?. Andrologia.

[42] Jurewicz J, Hanke W, Radwan M, Bonde JP (2009). Environmental factors and semen quality. Int J Occup Med Environ Health.

[43] de Angelis C, Galdiero M, Pivonello C, Salzano C, Gianfrilli D, Piscitelli P (2017). The environment and male reproduction: The effect of cadmium exposure on reproductive function and its implication in fertility. Reprod Toxicol.

[44] Jurewicz J, Radwan M, Sobala W, Polanska K, Radwan P, Jakubowski L (2015). The relationship between exposure to air pollution and sperm disomy. Environ Mol Mutagen.

[45] Okamura K, Kizu R, Toriba A, Murahashi T, Mizokami A, Burnstein KL (2004). Antiandrogenic activity of extracts of diesel exhaust particles emitted from diesel-engine truck under different engine loads and speeds. Toxicology.

[46] De Coster S, Van Larebeke N (2012). Endocrine-disrupting chemicals: associated disorders and mechanisms of action. J Environ Public Health.

[47] Alviggi C, Cariati F, Conforti A, De Rosa P, Vallone R, Strina I (2016). The effect of FT500 Plus(®) on ovarian stimulation in PCOS women. Reprod Toxicol.

[48] Rubes J, Selevan SG, Evenson DP, Zudova D, Vozdova M, Zudova Z (2005). Episodic air pollution is associated with increased DNA fragmentation in human sperm without other changes in semen quality. Hum Reprod.

[49] Madrigano J, Baccarelli A, Mittleman MA, Wright RO, Sparrow D, Vokonas PS (2011). Prolonged exposure to particulate pollution, genes associated with glutathione pathways, and DNA methylation in a cohort of older men. Environ Health Perspect.

[50] Bind MA, Lepeule J, Zanobetti A, Gasparrini A, Baccarelli A, Coull BA (2014). Air pollution and gene-specific methylation in the normative aging study: association, effect modification, and mediation analysis. Epigenetics.

