# Supplementary material for: Air pollution and female fertility: a systematic review of literature
Source: Reprod Biol Endocrinol. 2018 Dec 30;16:117. doi: 10.1186/s12958-018-0433-z (PMC6311303; doi:10.1186/s12958-018-0433-z)
Supplement: Supplementary file 1 — Table S1. Selection criteria according to PICO questions. (DOCX 14 kb) [file 12958_2018_433_MOESM1_ESM.docx]

**Table S1 Selection criteria according to PICO questions**

| Population | i. General population and IVF women | |
| --- | --- | --- |
| Intervention  Comparison | Exposure to air pollutants | |
| Outcomes | Fertility outcome |  |
| Study type | Clinical trials without time or language restrictions | |
